# Design and methodology of a mixed methods follow-up study to the 2014 Ghana Demographic and Health Survey

**DOI:** 10.1080/16549716.2017.1274072

**Published:** 2017-01-13

**Authors:** Sarah Staveteig, Richmond Aryeetey, Michael Anie-Ansah, Clement Ahiadeke, Ladys Ortiz

**Affiliations:** ^a^ Avenir Health, Glastonbury, CT, USA; ^b^ The Demographic and Health Surveys Program, Rockville, MD, USA; ^c^ University of Ghana School of Public Health, Accra, Ghana; ^d^ Center for Research and Review, Accra, Ghana; ^e^ Independent Consultant, Accra, Ghana; ^f^ ICF, International Health and Development Division, Rockville, MD, USA

**Keywords:** Mixed methods, follow-up survey, family planning, unmet need, computer-assisted personal interviewing, Demographic and Health Surveys

## Abstract

**Background:** The intended meaning behind responses to standard questions posed in large-scale health surveys are not always well understood. Systematic follow-up studies, particularly those which pose a few repeated questions followed by open-ended discussions, are well positioned to gauge stability and consistency of data and to shed light on the intended meaning behind survey responses. Such follow-up studies require extensive coordination and face challenges in protecting respondent confidentiality during the process of recontacting and reinterviewing participants.

**Objectives:** We describe practical field strategies for undertaking a mixed methods follow-up study during a large-scale health survey.

**Methods:** The study was designed as a mixed methods follow-up study embedded within the 2014 Ghana Demographic and Health Survey (GDHS). The study was implemented in 13 clusters. Android tablets were used to import reference data from the parent survey and to administer the questionnaire, which asked a mixture of closed- and open-ended questions on reproductive intentions, decision-making, and family planning.

**Results:** Despite a number of obstacles related to recontacting respondents and concern about respondent fatigue, over 92 percent of the selected sub-sample were successfully recontacted and reinterviewed; all consented to audio recording. A confidential linkage between GDHS data, follow-up tablet data, and audio transcripts was successfully created for the purpose of analysis.

**Conclusions:** We summarize the challenges in follow-up study design, including ethical considerations, sample size, auditing, filtering, successful use of tablets, and share lessons learned for future such follow-up surveys.

## Background

Nationally representative health surveys such as Demographic and Health Surveys, Multiple Indicator Cluster Surveys, and Performance Monitoring and Accountability 2020, are a rich source of information about vital statistics and health indicators in developing countries. The data produced by these surveys enable researchers and practitioners to measure, monitor, and evaluate self-reported health behaviors, characteristics, and outcomes in relation to biomarkers, geographic location, and social context. Yet the patterns in responses to standard questions posed in nationally representative health surveys are not always well understood. This is particularly true of attitudinal questions and prospective or retrospective questions translated into multiple languages and posed in varying cultural contexts.

Unmet need for family planning is an indicator measured through 18 separate questions posed to women in the Demographic and Health Surveys about sexual activity, fertility preferences, fecundity, and contraceptive use [1]. Married or sexually active fecund women who are not using contraception but who wish to postpone the next birth for two or more years or stop childbearing altogether are the primary group classified as having unmet need. Additionally, women who are pregnant or postpartum amenorrheic with an unwanted or mistimed pregnancy are considered to have unmet need. Among women with unmet need, survey reasons for not using contraception have been analyzed in detail [2]; this study aimed to ascertain the consistency and depth of stated reasons for non-use, gauge the stability of fertility intentions – previously shown to be fluid [3–5] – and to explore potential contraceptive misreporting, particularly traditional method use [6].

Meaningful insights into health behaviors and attitudes can be gained by conducting follow-up interviews with respondents, particularly semi-structured interviews where respondents have an opportunity to discuss the issue in an open-ended way [7]. Employing mixed methods helps overcome the weaknesses of either a purely qualitative or quantitative approach [8–10]. Samples derived through convenience, referral, and other approaches used in qualitative surveys are typically not generalizable [11]. The approach described herein, characterized as a sequential embedded design, is a mixed methods design that builds on the strength of the two-stage random sampling already undertaken for the main survey itself [12]. Provided that the study is conducted in a rigorous and systematic way, a small but diverse sample of follow-up respondents enables researchers to gauge the consistency of responses in a short timeframe and to better understand the meaning respondents ascribed to their original survey responses.

This article describes the methods used for the first ever mixed methods follow-up study embedded within a Demographic and Health Survey, focusing on unmet need for family planning. Researchers have called for mixed methods and qualitative studies on other topics to supplement and improve on other aspects of the Demographic and Health Surveys, such as pregnancy intentions, women’s empowerment, and gender-based violence [7,13–15]. Therefore, while the substantive findings from the study on unmet need are documented elsewhere [16], an in-depth standalone discussion of study design and implementation is expected to prove valuable for investigators fielding future such mixed methods follow-up studies.

## Methods

### Study setting

The study was carried out in Ghana, West Africa, in three purposively selected administrative regions: Greater Accra, Northern, and Central. The study was a follow-up to the 2014 Ghana Demographic and Health Survey (GDHS).

### Study design

The follow-up study was designed as a mixed methods study that followed up with a subset of respondents from a nationally representative survey, a model characterized as an embedded sequential mixed methods study [12] and described in detail by Schatz [8]. The follow-up study was funded, planned, and fielded independently from the main GDHS, but respondents were selected systematically from among the original GDHS respondents. Mixed methods studies are well-positioned to provide important insights about demographic behaviors; such studies are particularly valuable when open-ended responses can be compared against findings from large-scale population studies [7,10,17].

### The parent survey

The 2014 GDHS was a nationally representative survey of 9,396 women age 15–49 and 4,388 men age 15–59 residing in 11,835 interviewed households [18]. As with other Demographic and Health Surveys, the GDHS provides information on fertility, family planning, infant and child mortality, maternal and child health, nutrition, malaria, HIV, and non-communicable diseases in relation to respondents’ socioeconomic and demographic characteristics.

Fieldwork for the 2014 GDHS was conducted by the Ghana Statistical Service and the Ghana Health Service, with technical assistance from ICF International through The Demographic and Health Surveys (DHS) Program, which is funded by the United States Agency for International Development. As is standard with DHS protocol, the GDHS used a two-stage sampling design with probability proportional to sample size [19]. In total, 427 clusters from across the country were selected, and within each cluster 30 households were randomly selected for inclusion. The GDHS attempted to interview all women of reproductive age in each selected household. The household response rate was 98.5 percent, and among women age 15–49 in selected households the response rate was 97.3 percent [18].

### The follow-up survey

#### Recruitment of subjects

Three regions for follow-up were selected based on cultural and socioeconomic diversity, population size, and diversity in family planning use and fertility levels. These were Northern Region (NR), a very high-fertility region, Central Region (CR), a moderate-fertility region, and Greater Accra Region (GAR), with the lowest fertility. It was decided in advance of fieldwork that of the thirteen study clusters, five would be selected from NR, five from CR, and three from GAR. Within GAR all three clusters sampled would be urban, and within NR and CR there would be one urban and four rural clusters each. This ensured both a balance of urban and rural respondents and diversity among the urban population.

**Figure 1.**
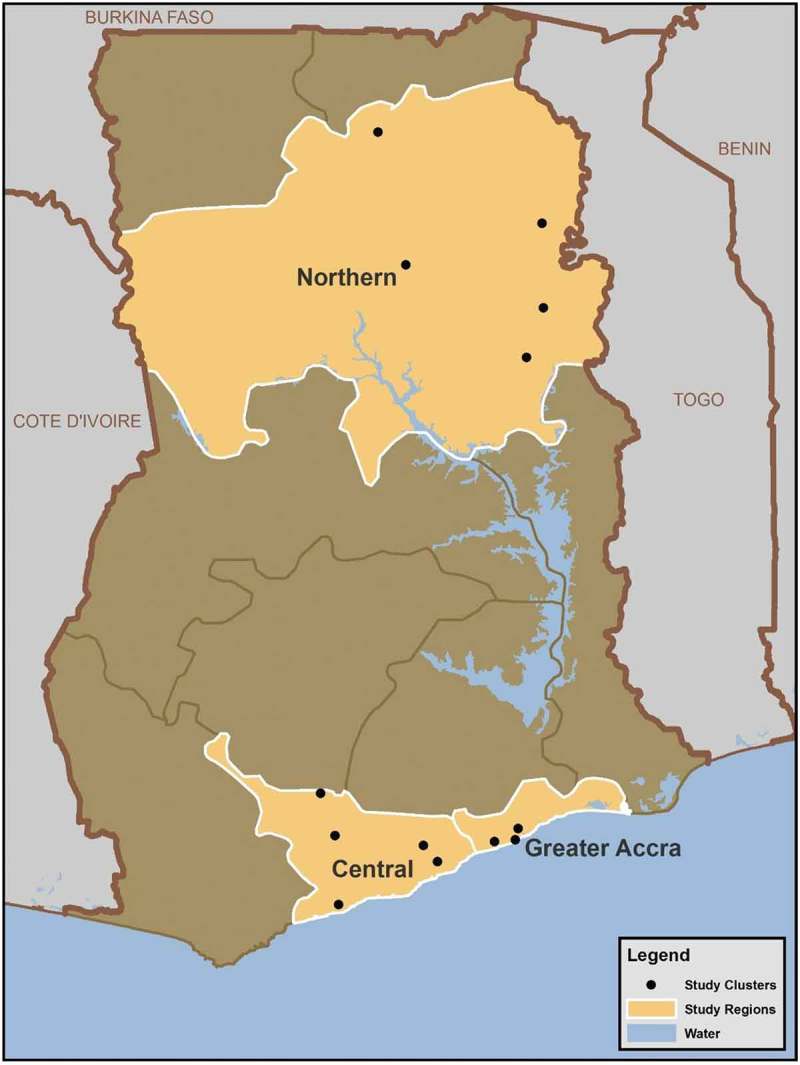
Survey clusters. Cluster locations illustrated on the map have been randomly displaced up to ten kilometers from their actual location using standard DHS procedures to ensure respondent confidentiality [20].

Within each region, clusters for the follow-up study were selected as the GDHS was being fielded. A completely random subsample of GDHS clusters would not have been feasible; cluster selection needed to balance diversity with logistical practicality. The GDHS fieldwork extended over several months, but the follow-up study was fielded by six interviewers working full-time over the course of a single month. The aim was to visit clusters for the follow-up study in October 2014 within one to four weeks of the date of initial interview in the GDHS. Hence only a limited number of clusters were available for selection. Ghana Statistical Service shared fieldwork team itineraries and progress reports with the follow-up study team. Based on an examination of these schedules, and reflecting a desire for geographic and cultural diversity, the follow-up study team proposed final cluster selections to ICF, which exercised some oversight for geographic diversity. The approximate locations of the final 13 clusters selected for the follow-up study are shown in Figure 1.

The 2014 GDHS used computer-assisted field editing (CAFE). Initial data entry was done in the field before the GDHS team moved to the next cluster. Paper questionnaires were sent to the Ghana Statistical Service office in Accra for validation. As the data for each of the 13 selected follow-up clusters arrived in the Ghana Statistical Service home office, and after the initial data entry had been validated, ICF staff used a CSPro program to confidentially select eligible follow-up respondents from among those who had consented to be contacted for a follow-up interview: married or sexually active women age 15–44 who either met the standard DHS definition of unmet need (excluding postpartum amenorrheic), or were in a subset of GDHS family planning users in the cluster [16]. The CSPro program automatically selected and output the variables and identification fields necessary for reinterview into a spreadsheet format for each eligible respondent; these were uploaded to a secure server that could be accessed by the implementing agency and downloaded to follow-up study tablets.

#### Data collection instruments

The follow-up study used three questionnaires, one for each of the eligible study groups: non-pregnant women classified by GDHS as having unmet need, pregnant women classified by GDHS as having unmet need, and a reference group of women currently using family planning. After an initial set of six identity verification questions, respondents were asked between one and three screening questions to confirm their eligibility for the assigned questionnaire (for example, ‘Are you currently doing something or using a method to delay or avoid getting pregnant?’). If the respondent indicated a different answer than was given to GDHS, for example because she had started or stopped using family planning since the last interview, interviewers were instructed to ask about the discrepancy and to switch to the correct questionnaire before proceeding.

The questionnaires covered topics such as reproductive intentions, family planning use, attitudes toward family planning, the role of partner and extended family in decision-making, and barriers to access. Respondents were also re-asked a few key questions about pregnancy, fertility preferences, family planning use, and reasons for non-use: first to ascertain consistency of responses, and second to allow interviewers to probe further into their meaning. In total the questionnaires contained between 24 and 31 groups of questions. A typical group of questions was comprised of an open-ended and closed-ended question, followed by a prompt to explain the closed-ended answer. The actual number of questions respondents were asked varied depending on the questionnaire type and on the skip pattern followed based on their own responses.

#### Interviewer training and pre-testing

An 11-day translation and interviewer training was conducted at the University of Ghana-Legon for eight interviewer training candidates. Training provided an overview of the study research questions and design, the concept of unmet need for family planning, and principles of qualitative interviewing. Field procedures were discussed extensively; interviewers learned how to use the audio recorders and Android tablets and engaged in back-translations of the questionnaires and in extensive role plays of interviews. Role plays provided an opportunity to test and revise the tablet program. The eight interviewer candidates and three field supervisors practiced how to download survey cases, upload results to the secure server, and change from one questionnaire type to another.

Interviewer training for the mixed methods follow-up survey was timed to coincide with GDHS fieldwork, such that the follow-up survey could conduct a pre-test in recently completed clusters in Accra toward the end of training. Pretesting in the two clusters involved running the selection program on the final data entry, collecting maps of the cluster and a field guide, and then seeing 12 respondents determined to be eligible. Each interviewer candidate conducted at least one pretest interview. The pretest proceeded through the follow-up survey process in full: requesting consent for interview and for audio recording, and administering a tablet-based interview with closed- and open-ended questions. Supervisors reviewed the downloaded data and audio and provided feedback to the interviewers; afterwards, slight revisions were made to the questionnaire and to the tablet program. At the end of training, all interviewers received copies of the final survey instruments and conducted full rehearsal interviews. Six of the eight interviewer candidates were hired for the study.

#### Fieldwork

Fieldwork for this study was conducted in October 2014. Three field teams, each consisting of two interviewers and a supervisor accompanied by a guide from the Ghana Statistical Service, tracked and identified the selected GDHS respondents, typically within one to four weeks of the original survey by using the household address, the name of the head of household, and the woman’s relationship to the head of household. In rural areas a village leader was approached for permission before beginning fieldwork. Interviewers returned to households up to three times to complete the interview. The questionnaires were implemented in Mobile Data Studio software on Android Samsung Galaxy tablets. Closed-ended responses were entered into tablets, and open-ended responses were captured using audio. The use of Computer-Assisted Personal Interviewing (CAPI) enabled answers to be compared in real time against responses given to the GDHS, and respondents could be asked about any discrepancies.

**Figure 2.**
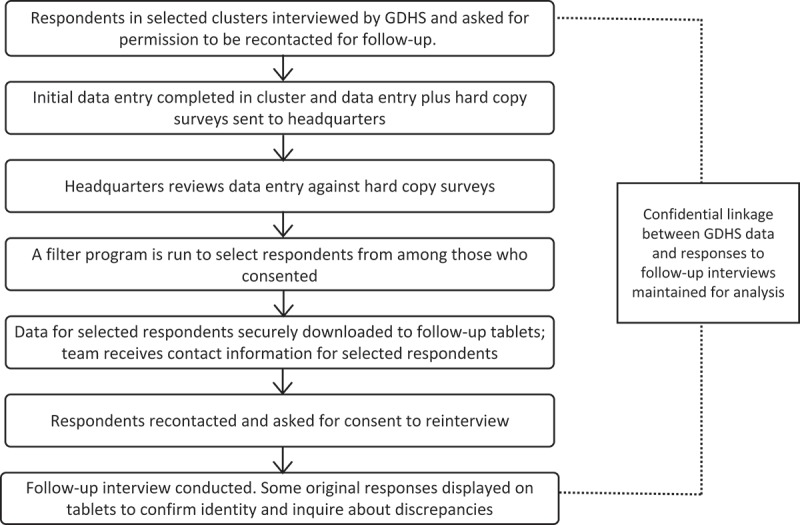
Overview of study procedure.

In order to confirm the identity of selected respondents and to enable the tablet to display appropriate GDHS data entry next to questions, a remote secure server had been set up to pre-populate data in follow-up questionnaires after selection of an eligible respondent and electronically signed verification (by the interviewer) that she had obtained the respondent’s consent to be interviewed. Respondents were asked six background questions to validate their identity: year of birth, month of birth, marital status, whether ever given birth, number of resident daughters, and number of resident sons. After the interview was completed and a field supervisor reviewed the tablet data entry, it was uploaded to the remote secure server and exported to a spreadsheet. Interviews were randomly audited by the Ghana Statistical Service guide to ensure that they were correctly completed. The entire process of fieldwork is summarized in Figure 2.

#### Response rate

Out of 9,396 total female respondents age 15–49 in the 427 GDHS clusters, 99.6 percent gave consent to be contacted again for the follow-up study. In the 13 clusters selected for the follow-up study, a data processing program determined 142 respondents to be eligible for follow-up based on their permission and the qualifying criteria discussed earlier. Of these, 135 women were successfully contacted and reinterviewed. Two women refused at the time of follow-up, and five were away or unable to schedule a follow-up interview despite repeated attempts.

Despite having matched on household address or structure number, name of head of household, and relationship to head of household, four of the 135 respondent identities could not be correctly verified during the data processing phase (for example there was a discrepancy in whether or not they had given birth). After excluding these unverified respondents, the final response rate was 92.3 percent. All respondents assented to audio recording of their interviews and the backup notebooks interviewers carried did not prove necessary.

Two of the respondents were daytime visitors to their household and thus had not been fully interviewed by the GDHS. They were excluded from the qualitative sample on the grounds that no information about family planning use had been gathered about them by the GDHS. The remaining 129 interviews were transcribed and, if necessary, translated into English. These resulted in over 1,000 pages of transcripts. Transcripts were entered into an ATLAS.ti database and systematically coded according to question number and theme. Additionally, the quantitative tablet data was cleaned and checked against the interview transcripts. These data were then imported into Stata and confidentially linked to the final DHS dataset for analysis.

#### Ethical clearance

Ethical clearance for the follow-up study was obtained in tandem with clearance for the GDHS from the ICF Institutional Review Board, which determined that both studies complied with all of the requirements of the US Code of Federal Regulations 45 CFR 46. Upon Institutional Review Board clearance, permission to share data between the GDHS and the follow-up study was obtained from the Ghana Statistical Service, which implemented the GDHS. As the GDHS was conducted on paper and cluster selection was not decided upon in advance, all female respondents age 15–49 were asked at the end of the GDHS questionnaire, in the language of their interview, if they would consent to be re-contacted for a follow-up study on family planning and childbearing. The follow-up survey obtained consent prior to reinterview, both for the interview itself and for audio recording of responses.

In keeping with Institutional Review Board regulations, the confidentiality of the respondent’s information was maintained at all stages of the survey. Anonymous cluster and respondent identifiers were created and used for recordkeeping. The original information used to locate respondents for follow-up (name of household head, address, and all initial data entry from the GDHS) was destroyed by interviewers and supervisors at the conclusion of fieldwork, and only a new, anonymized identification number was maintained for correspondence with the DHS home office.

Following the conclusion of the GDHS, cluster and household numbers for all respondents nationwide were scrambled according to established DHS protocol, and original records of cluster and household numbers were destroyed prior to linking respondents’ information with HIV test results. With permission from Ghana Statistical Service and the ICF Institutional Review Board, the follow-up study was able to maintain an internal, confidential linkage between the anonymously identified follow-up respondents and the final, scrambled GDHS dataset.

## Results and discussions

### Lessons learned

This study was the first mixed methods follow-up study conducted by the Demographic and Health Surveys Program since its inception more than 30 years ago. Several methodological lessons were learned during the course of data collection that may benefit future embedded follow-up studies.

In addition to asking open-ended questions about the topic, it was also useful for the follow-up study to ask some of the same closed-ended questions asked in the parent survey. Access to the respondent’s original answers to key questions through data exported from the original interview was essential to the study in two respects. First, it enabled systematic selection of respondents based on a complex algorithm to determine eligibility. Second, original responses could be displayed on interviewer’s tablets, allowing any discrepancies to be diplomatically discussed with respondents. The reasons for discrepancies can be very useful from a data-quality perspective (e.g. did the respondent misunderstand a DHS question the first time around or did her situation change in the interim), as well as from a substantive perspective (did fertility intentions change because they were ambivalent to begin with, was there a particular circumstance that changed her mind, or does she think there was a mistake in earlier data input). Anticipating possible discrepancies and programming appropriate skip patterns was an important part of survey preparation.

Unfortunately, despite training, interviewers did not ask about some discrepancies during the follow-up interviews for this study. They reported confusion with the process of changing to a different questionnaire when respondents reported discrepant family planning use, and had prioritized preserving rapport over noticing and asking about discrepancies. While rapport is essential, improvements in skip pattern design and in tablet display (for example, only one screen per question) would have yielded more explanations for discrepancies.

Future follow-up studies could consider importing original data into the tablets but not displaying it unless there was a discrepancy. This would help guard against – though not completely preclude – any possible collusion to produce inflated estimates of data quality. In this survey, there was an audio recording of the exchange between interviewers and respondents plus auditing of fieldwork by Ghana Statistical Service, so it was not deemed necessary to hide the original responses. Future studies without an audio component or an outside auditor could consider an additional level of identity verification, particularly if the survey software was able to flag discrepant responses, despite any subsequent correction.

The 2014 GDHS was conducted with paper questionnaires. Data entry was done in the field using the CAFE system and finalized in the central office after paper questionnaires were received. While the CAFE system improves the speed of data entry substantially, there was necessarily at least a few days between fieldwork and finalized, checked electronic data entry received in the home office for the cluster as a whole. The data entry was needed in order to filter and select respondents and to export data fields to follow-up tablets. The complexity of the algorithm to determine eligibility for unmet need caused a glitch that resulted in over-identification of respondents which was not known at the time of fieldwork. It is easy enough to advise additional checks on the filtering program for future surveys; more importantly, simpler selection criteria may be warranted. Simplifying selection criteria may be helpful if it produces additional reference groups, but the obvious risk is dilution of the target group for interviews; in this case, women with unmet need for family planning.

The follow-up fieldwork faced several challenges. It was frequently difficult to find households that had been selected for reinterview. In areas without street names and numbers, GDHS listing teams typically painted structure numbers onto households; in the interim between the two surveys, many numbers had washed away or been painted over. Interviewers had to ask several neighbors to locate the correct household; this was particularly difficult in clusters where households were numbered in a serpentine pattern and difficult to locate.

Additionally, because the GDHS interview data were collected on paper and identifying information was not included in data entry, the follow-up study did not have access to women’s names. Identifying respondents for the follow-up study relied on the name of the household head and the relationship to the household head recorded in the GDHS. Proper identification of respondents occasionally proved confusing when, for example, there were multiple wives or multiple daughters of the household head. In these cases, original respondents within the same household were differentiated based on year of birth, marital status, or number of resident children.

Due to strict ethical protocols for the GDHS as a whole, it was not possible to gather additional information typically used to recontact respondents in longitudinal surveys, such as telephone numbers or individual household GPS coordinates. Future sub-studies may consider linking to a parent survey where it would be possible to gather the respondent’s telephone number to simplify the process of scheduling a follow-up interview. Even a low-tech return identifier like leaving a card with a number on it could be helpful for reconfirming identity.

Barring additional means of re-identification, future mixed methods follow-up studies could consider fieldwork during a survey that uses CAPI, which would enable automatic identification of eligible respondents at the end of a given interview. Permission could be immediately requested from only those eligible for the follow-up study. Data required for the reinterview could be securely transmitted via Bluetooth to a follow-up interviewer’s tablet and the respondent could be found much more easily. Initially, interviewee fatigue was a concern, but the follow-up study took only 20–30 minutes on average; a one-day gap between interviews as currently happens for verbal autopsy modules may in fact be no more taxing than a two-week gap.

## Conclusion

Embedding semi-structured follow-up interviews within the larger GDHS survey had a number of advantages. First, the study benefited from the rigorous and standardized sampling and household listing process undertaken by DHS surveys. A random selection of respondents within each cluster enabled targeting of women who do not use family planning and thus would not ordinarily be reached by the kind of convenience sampling that typically takes place within or outside health facilities. Current family planning users were included as a reference group. Second, the information already gathered about respondents aided the collection of additional data. Interviewers did not need to repeat the extensive battery of questions that had already been posed by the GDHS. Third, by interviewing original respondents of a large-scale survey, the study had the opportunity to assess the consistency of information provided and to link such data to nuanced qualitative data about respondents’ lived experiences to the quantitative survey results. In doing so, it provided insight about narratives and rationale not evident from quantitative data.

Overall, this study demonstrates a feasible strategy for building upon established nationally representative health surveys to shed light on important issues underlying observed trends in maternal and reproductive health. Intensive data from the small follow-up sample found an ambivalence in women’s prospective fertility intentions, significant underreporting of traditional methods, a systematic omission of abstinence as an intentional method of family planning, and more substantial opposition to modern methods than recorded in the survey itself [16]. These are valuable insights into the complexity of unmet need for family planning in Ghana and help to increase our understanding of findings from the GDHS. They deserve additional explorations in other national contexts as well. We recommend embedded follow-up studies on other topics and in other countries as a periodic tool to assess survey data quality, to improve questionnaire design, and to understand how well respondents’ lived experiences accord with conclusions drawn from closed-ended survey responses.

## Paper context

Qualitative data provide substantial insight into health behaviors, but are rarely generalizable. Linking qualitative and mixed methods data to nationally representative data from large-scale surveys adds value to quantitative studies. This article reports on an innovative mixed methods follow-up study about unmet need conducted among a systematically selected subset of respondents to the 2014 Ghana Demographic and Health Survey. We share challenges and successes in design and implementation that may benefit future such follow-up studies.
